# New Electrochemical Sensor Based on Hierarchical Carbon Nanofibers with NiCo Nanoparticles and Its Application for Cetirizine Hydrochloride Determination

**DOI:** 10.3390/ma15103648

**Published:** 2022-05-20

**Authors:** Anna Górska, Marcel Zambrzycki, Beata Paczosa-Bator, Robert Piech

**Affiliations:** Faculty of Materials Science and Ceramics, AGH University of Science and Technology, Al. Mickiewicza 30, 30-059 Krakow, Poland; agorska@agh.edu.pl (A.G.); zambrzycki@agh.edu.pl (M.Z.); paczosa@agh.edu.pl (B.P.-B.)

**Keywords:** voltammetry, metal nanoparticles, cetirizine, glassy carbon electrode, carbon nanofibers, carbon nanotubes

## Abstract

A new electrochemical sensor based on hierarchical carbon nanofibers with Ni and Co nanoparticles (eCNF/CNT/NiCo-GCE) was developed. The presented sensor may be characterized by high sensitivity, good electrical conductivity, and electrocatalytic properties. Reproducibility of its preparation expressed as %RSD (relative standard deviation) was equal to 9.7% (*n* = 5). The repeatability of the signal register on eCNF/CNT/NiCo-GCE was equal to 3.4% (*n* = 9). The developed sensor was applied in the determination of the antihistamine drug—cetirizine hydrochloride (CTZ). Measurement conditions, such as DPV (differential pulse voltammetry) parameters, supporting electrolyte composition and concentration were optimized. CTZ exhibits a linear response in three concentration ranges: 0.05–6 µM (*r* = 0.988); 7–32 (*r* = 0.992); and 42–112 (*r* = 0.999). Based on the calibration performed, the limit of detection (LOD) and limit of quantification (LOQ) were calculated and were equal to 14 nM and 42 nM, respectively. The applicability of the optimized method for the determination of CTZ was proven by analysis of its concentration in real samples, such as pharmaceutical products and body fluids (urine and plasma). The results were satisfactory and the calculated recoveries (97–115%) suggest that the method may be considered accurate. The obtained results proved that the developed sensor and optimized method may be used in routine laboratory practice.

## 1. Introduction

Cetirizine hydrochloride belongs to the group of the second-generation H_1_ antihistamines. It may be characterized by high specificity for the H_1_ receptors. In contrast to first-generation antihistamines, CTZ does not cause such severe drowsiness. It is considered safe and can be taken over a long period of time, which was proven during the clinical trials [1,2,3]. CTZ is rapidly absorbed (maximum concentration in plasma achieved in ~1 h after oral administration), and most of the dose is excreted in an unchanged form [2,3,4].

Various analytical methods may be used for the determination of CTZ, among them: high performance liquid chromatography (HPLC) [5,6,7], liquid chromatography (LC) [8,9], spectrophotometry [10,11], capillary electrophoresis [12,13], and voltammetry [14,15,16,17,18,19,20,21,22,23,24]. Although the methods mentioned can be applied successfully in the determination of CTZ, some of them may suffer from several disadvantages. They require toxic solvents and produce a lot of waste, a high amount of the sample is necessary for single analysis, the sample preparation process is complicated and time consuming, and in the case of some methods, cost of analysis is relatively high. Voltammetry is an example of the method that allows for the avoidance of those inconveniences. It is an electrochemical method characterized with high sensitivity and a low-detection limit. The most important part of each voltammetric system that determines the performance of the developed method is the working electrode (WE). Different constructions and materials may be used to develop the WE. A current trend in analytical chemistry is focused on the development of new types of solid electrodes, among them, the glassy carbon electrode (GCE) and the carbon paste electrode (CPE). Those two types of sensors may be characterized by good chemical and mechanical properties, wide-working potential range and what is the most interesting—the possibility of their modification. The modification of the GCE and CPE allows to improve parameters, such as selectivity, sensitivity, and limit of detection; therefore, such modified sensors are willingly used in electrochemical analysis. Developed modifiers should exhibit certain properties: good electrical conductivity, high-surface area, electrocatalytic properties, and good mechanical and chemical resistance. Materials that may be used in such applications often belong to the group of carbon nanomaterials (carbon black, multi- or single-walled carbon nanotubes, graphite) [25,26], metal particles (Au, Pt, Ni) [27,28], metal oxides (RuO_2_, TiO_2_) [29,30], conducting polymers (PEDOT, polyaniline) [31,32], and many others. Hybrid materials made of two or more different components have also become popular. It allows for the combination of features of each component, which often results in better properties of the developed sensor. Some of the most interesting hybrid materials are hierarchical nanocomposites of carbon nanotubes grown directly on the metallic nanoparticles embedded on the surface carbon nanofibers. Such modification is especially advantageous due to the presence of carbon nanotubes and metallic nanoparticles, which significantly increases the density of active sites, ensures the extended electrical conduction pathway, and prevents the agglomeration of nanotubes. To best of our knowledge, there are no reports of application of such nanostructures in voltametric determination methods.

The literature reports that CTZ was determined using voltammetric techniques and different types of solid electrodes: the boron-doped diamond electrode [19], the graphite pencil electrode [20], and the modified GCE [15,17,21,23] and CPE [14,16,18,22,24].

The aim of this work was to develop a new type of electrochemical sensor based on a novel hierarchical nanocomposite—carbon nanofibers/carbon nanotubes/NiCo nanoparticles (eCNF/CNT/NiCo). The nanocomposite was characterized by favorable properties, such as good electrical conductivity, large surface area ensuring multiple absorption active sites, and high-electrocatalytic activity. The sensor was obtained through modification of the GCE with ink prepared from the eCNF/CNT/NiCo nanocomposite, and it was used in the determination of the antihistamine drug—cetirizine.

## 2. Experimental

### 2.1. Apparatus

#### 2.1.1. Characterization of Nanocomposite

Observations of the microstructures of nanocomposites were made using the scanning electron microscope (SEM), Nova NanoSEM 200 (FEI Europe Company, Hillsboro, OR, USA), at an acceleration voltage of 10 kV. The high-magnification imaging was carried out using the transmission electron microscope, (TEM) FEI Tecnai TF20 X-TWIN (FEG), at 200 kV. The TEM observations were performed in the bright field and high-resolution modes.

The grazing incidence X-ray diffraction (GIXRD) measurements were carried out using the Panalytical X’Pert Pro X-ray diffractometer (PANalytical, Malvern, UK) equipped with Cu Kα X-ray source (λ = 1.5406 Å). Diffractograms were collected in the 2θ range from 15 to 65° with a step of 0.02°. Analysis of the obtained diffraction patterns was performed using X’Pert HighScore Plus 2.0 (PANalytical, Malvern, UK), and Fityk 0.9.8 software (M. Wojdyr). The interplanar distance between the graphene layers, d_002_, and mean size of graphitic domains in the *c*-axis direction, L_c_, were estimated using Bragg’s and Scherrer’s equations, respectively [33].

Raman spectroscopy measurements were performed at LabRAM HR spectrometer (Horiba, Ltd., Kyoto City, Japan) using 50× objective and 532 nm laser excitation source. The covered spectral range was from 50 to 4000 cm^−1^ with resolution of 0.39 cm^−1^. A total of five accumulations with 10 s integration time were recorded for each spectrum. Data was processed using Fityk 0.9.8 software, and deconvolution of the complex bands was made using the PseudoVoight function.

The electrical conductivity of hybrid nanofibers was examined using T2001A3 four-point probe system (Ossila Ltd., Sheffield, UK). The distance between the probes was 1.27 mm, and the target current was set at 10 mA. A total of 25 separate runs were recorded and averaged. The measurements were carried out on the rectangular thin sheets (8 × 15 mm) directly cut from the nonwovens.

#### 2.1.2. Analytical Measurements

The multipurpose electrochemical analyzer, M161, and the electrochemical stand, M164 (both mtm-anko, Poland), were used for all voltammetric measurements. A typical three-electrode system was used: working electrode—eCNF/CNT/NiCo-GCE; reference electrode—silver; silver chloride in 3 M KCl; and auxiliary electrode—platinum wire. The electrodes were submerged in a quartz cell (total volume of 20 mL) filled with supporting electrolyte (10 mL). The solution in the quartz cell was stirred using a magnetic stirrer and a rotating bar (~500 rpm). All pH measurements were carried out using laboratory pH-meter (N-512 elpo, Polymetron, Wroclaw, Poland). Sonication of suspensions and solvents was carried out using an ultrasonic bath (Intersonic IS-1K, Olsztyn, Poland).

### 2.2. Chemicals and Glassware

All chemicals used were of analytical grade and were used without further purification. The standard solution of cetirizine hydrochloride (0.01 M) was prepared by dilution of the appropriate amount of reference material (Sigma Aldrich, Darmstadt, Germany) in double distilled water; after preparation, it was stored in the refrigerator at 4 °C. The solutions with lower concentrations were prepared daily by dilution of the standard solution. Lyophilizate urine (Medidrug^®^ Basis-line U) was purchased from Medichem, Steinenbronn, Germany, and the human plasma was purchased from Biowest, Nuaillé, France. The supporting electrolyte solution (phosphate buffer pH 7.0) was prepared by mixing KH_2_PO_4_ and K_2_HPO_4_ (both Merck, Darmstadt, Germany) in appropriate proportions. The remaining reagents were purchased as follows: polyacrylonitrile (PAN; M_w_ = 150,000 Da)—MilliporeSigma, Darmstadt, Germany; PAN-terpolimer (5% acrylane methyl and 1% of alilosulfonian mers content)—Zoltek, Nergez, Hungary; nickel acetylacetonate (Ni(Acac)_2_) and cobalt acetylacetonate (Co(Acac)_2_)—Acros Organics, Belgium; hydrogen and nitrogen—Air Liquide, France; dimethylformamide (DMF) and methanol—POCH, Gliwice, Poland; Al_2_O_3_—Buehler Micropolish, Lake Bluff, IL, USA; Triton X-100, potassium chloride, microcrystalline cellulose, caffeine, lactose monohydrate, aspartame, magnesium stearate, trichloroacetic acid (TCA)—Sigma Aldrich, Darmstadt, Germany. All glassware has been cleaned using HNO_3_ solution and double-distilled water before use. For preparation of all solvents used, as well as for measurements, double-distilled water was utilized.

### 2.3. Sample Preparation

#### 2.3.1. Tablets

For the measurement of CTZ in tablet formulations, Allertec WZF (10 mg CTZ in tablet) and Cirrus Duo (5 mg of CTZ) were used. First, three tablets of each medication were weighed and then crushed in a mortar. Appropriate amounts of each sample were weighed, transferred to the beaker (25 mL), and dissolved in 15 mL of double-distilled water (for intensification of the process, the beaker was placed in the ultrasonic bath for 10 min). The solutions obtained were filtrated using syringe cellulose filters (pore size 0.2 µm), transferred to the volumetric flask (25 mL volume) and filled up to the mark with double-distilled water.

#### 2.3.2. Lyophilizate Urine

For the measurements of the CTZ concentration in urine, lyophilizate urine (Medidrug^®^ Basis-line U, Steinenbronn, Germany) was used. Before measurement, the sample in the vial was dissolved in 5 mL of double-distilled water, as suggested by the producer. In the next step, the sample was filtrated using a syringe filter (pore size 0.2 µm) and appropriately diluted with the supporting electrolyte solution.

#### 2.3.3. Plasma

The purchased plasma (Biowest, Nuaillé, France) was stored in a freezer at the temperature of −20 °C (as suggested by a producer) and was defrosted directly before measurement. To remove protein matrix components that may interfere during voltammetric measurements, 800 µL of plasma was mixed with 200 µL of 10% TCA. In the next step, the mixture was shaken for 2 min and then centrifuged for 30 min. The supernatant was collected and filtrated using a syringe filtrate (pore size 0.2 µm). The sample was prepared according to the described procedure, was appropriately diluted with supporting electrolyte, and was measured using the developed method.

### 2.4. Synthesis of Nanocomposite

Modifier nanocomposite was obtained in the process combining the electrospinning of precursor nanofibers, high-temperature heat treatment, and catalytic CVD synthesis of carbon nanotubes directly on the surface of eCNF. The spun solutions for electrospinning contained 11.5% of 2:1 mixture of pure PAN, and PAN-terpolymer, 1.5% of Ni(Acac)_2_, and 1.5% of Co(Acac)_2_ in DMF. The precursor nanofibers were spun using a custom-made electrospinning device with rotational collector, and a syringe with the gravitational outflow. The conditions for electrospinning were as follows: applied voltage—9 kV; nozzle-collector distance—40 mm; nozzle diameter—1.1 mm; temperature—30 °C; humidity in the chamber ~15%; and duration—30 min. The as-spun nonwovens were subjected to the thermal stabilization treatment in the atmosphere of air, involving heating up from RT to 240 °C, annealing in this temperature for 30 min, ramping up to T = 260 °C, and then annealing for the next 20 min. The heating rate during the stabilization was 3 °C min^−1^. During this process various chemical reactions took place, including the cyclization, oxidation, and dehydrogenation of PAN and the thermal decomposition of Me_x_(Acac)_y_. The main purpose of stabilization was to make the polyacrylonitrile non-meltable for further processing, to increase the carbon yield, and to decompose the acetylacetonates into the metal oxides. Next, the cooled nanofibers were transferred into the tubular reactor where they were heated up to 650 °C in the continuous flow of N_2_. After reaching this temperature, the H_2_/N_2_ mixture was introduced into the chamber (Q = 130/50 mL min^−1^) with an aim of reducing the Me_x_O_y_ and their conversion into metallic nanoparticles. After 80 min, the temperature was increased up to 775 °C, and the gas flow was replaced with a mixture of C_2_H_2_/N_2_ (Q = 15/450 mL min^−1^). At this stage, the presence of NiCo nanoparticles induced catalytic growth of carbon nanotubes, and this process was carried out for the next 10 min. Finally, to fully carbonize the nanofibers, the pure N_2_ flow was restored and the temperature in the furnace was raised up to 1000 °C and held for 20 min. After cooling down, the nonwoven was crushed in the mortar and mixed with DMF to obtain ink with a concentration of 1 mg ml^−1^. The suspension was sonicated with an ultrasonic processor (Vibra-cell VCX 130, Newtown, CT, USA, P = 130 W, f = 20 kHz) for 5 min with 45% of power, and then for 5 min with 30% of power. The obtained dispersion was used for the further modification of the GCE.

### 2.5. Working-Electrode Preparation

The modified sensor was prepared using a typical glassy carbon electrode (GCE) with a 3 mm diameter. Before modification, the GCE was properly prepared to remove contamination and to smooth the electrode’s surface. In the first step, the electrode was polished using Al_2_O_3_ slurry (particle size 0.3 µm) until smooth and a shiny surface of the glassy carbon was obtained. Then, the electrode was rinsed in the stream of double-distilled water and placed in a methanol–water mixture in an ultrasonic bath for approximately 3 min. The cleaned electrode was allowed to dry, and then it was ready to be modified. The modification process was performed as follows:

- Homogenization of modifier suspension—ultrasonic bath for 45 min (every 15 min, the water in the bath was changed to fresh water to prevent overheating of the suspension);
- Application of the modifier suspension (7.5 µL) on the surface of the prepared GCE;
- Evaporation of the solvent from the modifier—approximately 3 h.

After the described preparation process, the modified sensor was ready to use in the voltammetric measurements.

### 2.6. Measurement Procedure

For quantitative measurements of CTZ in real samples, differential pulse voltammetry (DPV) and the standard addition method were used. Measurements were carried out in the supporting electrolyte consisting of 0.1 M phosphate buffer (pH 7.0). Before the first use, the working electrode was stabilized by performing approximately 30 measurements. The instrumental parameters of DPV were set as follows: sampling and waiting time t_s_ = t_w_ = 10 ms (step time 20 ms), step potential E_s_ = 6 mV, and pulse amplitude ΔE = 75 mV. Further steps were taken according to the following procedure:

- Cleaning of the electrode surface: E = −1200 mV; t = 10 s;
- Accumulation step: E_acc_ = 300 mV; t_acc_ = 10 s;
- Rest period: t = 3 s;
- Voltammograms registration in the potential range 300–1200 mV.

## 3. Results

### 3.1. Characterization of Nanocomposite

**Figure 1:**
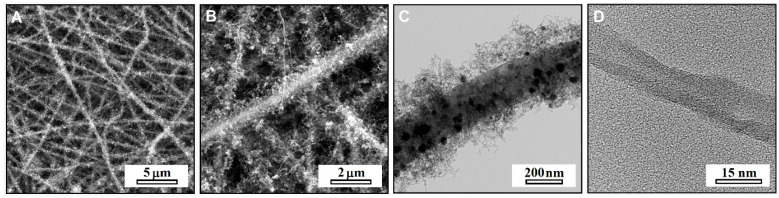
SEM (**A**,**B**) and BFTEM (**C**) images of hierarchical nanofibers—eCNF/CNT/NiCo. (**D**) HRTEM close-up of MWCNT.

SEM and TEM images of the obtained nanocomposite are shown in Figure 1. The observations confirmed the successful formation of the hierarchical-fibrous morphology and did not show any significant microstructural defects of material. The carbon nanotubes grown on the NiCo nanoparticles homogeneously covered the entire surface of the core nanofibers, thus ensuring the large surface area and high density of the electrochemically active sites. The growth mechanism of CNT involved both tip- and base-mechanisms, while the increasing duration resulted in increases of the lengths of the nanotubes. As revealed by HRTEM, the nanotubes had multiple walls (MWCNT) and were characterized by strong disorder manifested by a large number of defects and non-parallelism of the walls. The mean diameters of the core nanofibers, carbon nanotubes, and NiCo nanoparticles were: d_eCNF_ ~ 250 nm, d_CNT_ ~ 15 nm, and d_NiCo_ ~ 15 nm. The EDS spectrum of the nanocomposite was characterized by the presence of emission lines from carbon, oxygen, nickel, cobalt, and copper (Supporting Information, Figure 1). The presence of oxygen should be attributed to the functional groups and the defects on the surface of eCNF, which are a normal feature of carbon nanomaterials. In turn, the signal from copper originated from the used TEM support grid and was not related to the composition of the sample itself. The HRTEM image showing the NiCo nanoparticles can be found in the Supporting Information—Figure S2. The measurements of the interplanar distance in NiCo showed the value of d = 0.205 nm, which perfectly matches the (111) NiCo atomic planes distance.

**Figure 2:**
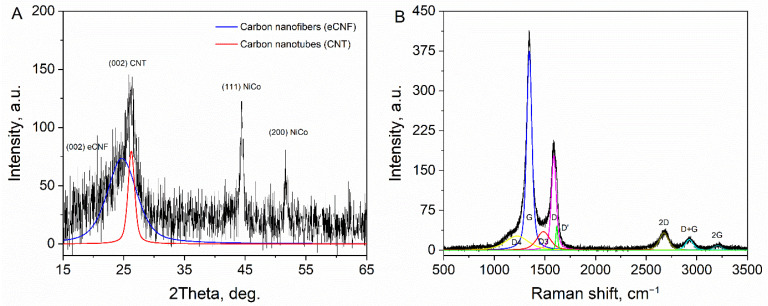
(**A**) GIXRD diffraction pattern and (**B**) Raman spectra of hierarchical nanofibers, eCNF/CNT/NiCo.

The GIXRD pattern of the nanocomposite fibers is shown in Figure 2A. The most prominent, broad feature at 2θ ~ 25° originates from the overlapping of the reflexes from (002) planes of eCNF and CNT. The L_c_ values calculated from these lines were 6.01 nm (CNT) and 1.40 nm (eCNF), while the interplanar distances between the neighboring graphene planes, d_002_, were 0.340 nm (CNT), and 0.363 nm (eCNF). Differences in these values demonstrate the better structural ordering and crystallinity of the obtained CNT as compared to the core-eCNF. The lack of reflexes from the other carbon planes shows the disruption of the 3D long-range ordering and indicates the turbostratic structure of both carbon phases. The presence of diffraction peaks at 44.7° (111) and 51.7° (200), evidence of the successful reduction of metal oxides and the formation of NiCo alloy nanoparticles (JCPDS; Ni: 15-0806, Co: 01-1260). The value of the mean crystallite size estimated from the (111) NiCo line was 12.40 nm, which indicates the predominantly monocrystalline character of nanoparticles.

The Raman spectra of the nanocomposite (Figure 2B) comprised of several first- and second-order bands carrying information about the internal structure of carbon. The first-order spectrum were resolved with the 5-band model containing the following bands: G line (~1580 cm^−1^) originating from the E_2g_ stretching of sp^2^ C=C bonds; D resonance band (~1360 cm^−1^) related to transitional defects of the graphitic lattice; D’ shoulder line (~1620 cm^−1^) sensitive to the type of defects; D4 (~1200 cm^−1^) and D3 (~1500 cm^−1^) components, both related to the hydrogenated and disordered carbon. The more distant spectrum comprised of 2D, D + G, and 2G bands, representing the overtones of the first order bands. The shape of the spectra and appearance of the mentioned bands indicate a moderate structural disorder characteristic for CNT and eCNF. The ratio of integral intensities of D and G bands—a common parameter describing the defectiveness of the carbon structure—was I_D_/I_G_ = 2.26. The numerous connections between the eCNF and CNT, together with the high concentration of π-electrons from sp^2^ carbon ensured a highly efficient pathway for conduction of the electrical current. The four-probe measurement revealed conductivity of nanofibers, σ = 7.97 S cm^−1^.

### 3.2. Sensors Characterization

#### 3.2.1. Volume of Surface Modifier

**Figure 3:**
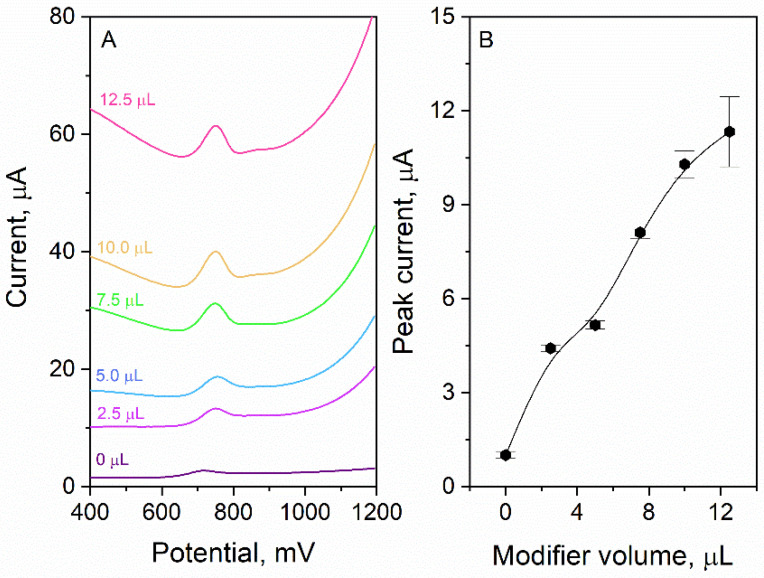
Dependence of the volume of applied surface modifier on the CTZ signal. Experiment conducted in 0.1 M phosphate buffer, CTZ concentration was equal to 10 µM. (**A**) DPV voltammograms, (**B**) graph.

The influence of volume of surface modifier on CTZ signal was investigated. During the experiment, the GCEs were modified with the following volumes of the modifier: 0; 2.5; 5.0; 7.5; 10.0; 12.5 µL. The experiment was carried out in 0.1 M phosphate buffer (pH 7.0); the CTZ concentration was equal to 10 µM. As it was presented in Figure 3, the higher was the modifier volume, and the higher was the CTZ peak current. When no modifier was applied (bare GCE), the current response was relatively low and was equal to 1.02 µA. The maximum peak current was achieved for the volume 12.5 µL—11.3 µA. Nevertheless, it is worth noting that for volumes of 10.0 µL and 12.5 µL, the error bars are high, which means that the register signal is not stable (RSD for 10.0 µL—4.7% (*n* = 9) for 12.5 µL—16.3% (*n* = 9)). Additionally, it may be observed that, the higher was the volume of surface modifier, and the higher was background current. Considering the presented arguments, we decided that the optimal volume of the surface modifier is 7.5 µL; the peak current was relatively high (8.1 ± 0.2 µA), and the signal was stable (RSD = 3.0%) with an acceptable level of the background current. The improved response of the sensor after the modification with eCNF/CNT/NiCo originates from multiple active sites provided by the CNT/NiCoNP interface, while the main role of eCNF was to provide a hierarchical conduction pathway and to prevent the agglomeration of nanotubes and metallic nanoparticles.

#### 3.2.2. The Reproducibility and Repeatability of the Sensor

To assess the reproducibility of the sensor preparation, five GCEs were modified with 7.5 µL of surface modifier. In the next step, the signal derived from 10 µM of CTZ was measured on each electrode. To evaluate how electrodes differ from each other, the RSD parameter was calculated based on the obtained results. Its value was equal to 9.7% (*n* = 5), which suggests good reproducibility of sensor preparation. To check the repeatability of the sensor work, one from the prepared modified electrodes was chosen for the experiment. Then, the CTZ signal was registered nine times and based on the obtained peak current values, RSD was calculated. It was equal to 3.4% (*n* = 9, CTZ concentration 1 µM), which suggests good repeatability of the sensor’s work.

### 3.3. Cetirizine Behavior on the Surface of eCNF/CNT/NiCo-GCE

**Figure 4:**
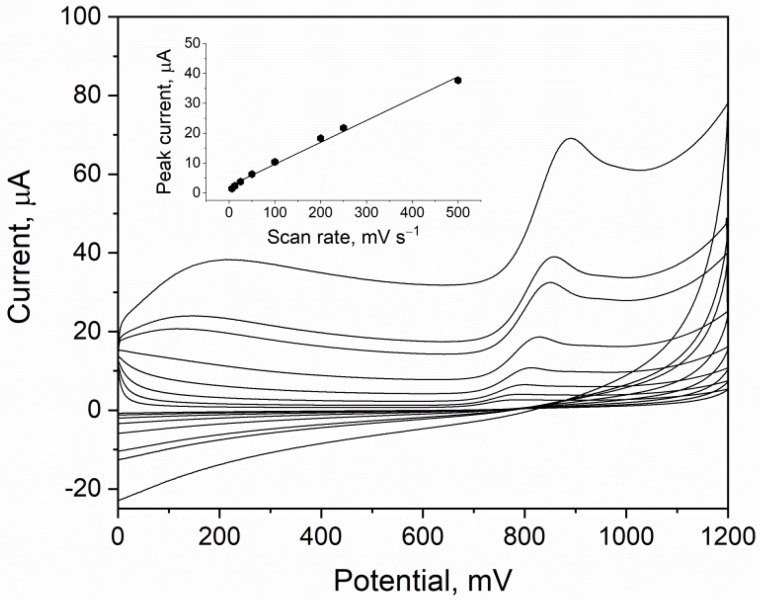
CV LSV measurement of 100 µM of CTZ in 0.1 M phosphate buffer. Scan rate values were in the range of 6.3 mV s^−1^ to 500 mV s^−1^. Dependence of CTZ peak current on scan rate (inset).

The linear sweep voltammetry (LSV) technique was used to characterize CTZ behavior on the eCNF/CNT/NiCo-GCE sensor. The experiment was carried out in 0.1 M phosphate buffer (pH 7.0), and the CTZ concentration was equal to 100 µM. Voltammograms were registered for different values of the scan rate from the range of 6–500 mV s^−1^. For each scan rate value, the anodic peak derived from CTZ oxidation is visible (Figure 4). It suggests an irreversible character of the CTZ electrode reaction. When the scan rate value was increased, a shift in the peak potential toward more positive values was also noticed. It may also be observed that the CTZ peak current was increasing with the scan rate value. The acquired data were used to determine the diffusion or adsorptive character of the electrode process. For this purpose, the dependences of the CTZ peak current on scan rate and square root of scan rate were plotted. A linear relationship was obtained for scan rate dependence (*r* = 0.991) (inset of Figure 4), which suggests an adsorptive character of the electrode process. To bring closer the mechanism of the CTZ reaction, the dependence of the peak potential on the natural logarithm of the scan rate was plotted. The linear dependence was obtained with the slope (b) equal to 0.026. Using the Laviron’s equation for the adsorption-controlled processes (b = RT/αnF), it was calculated that αn was equal to 1.02. After assuming α = 0.5, it can be calculated that the number of electrons (*n*) that participate in the reaction of the CTZ electrode is equal to 2.

**Figure 5:**
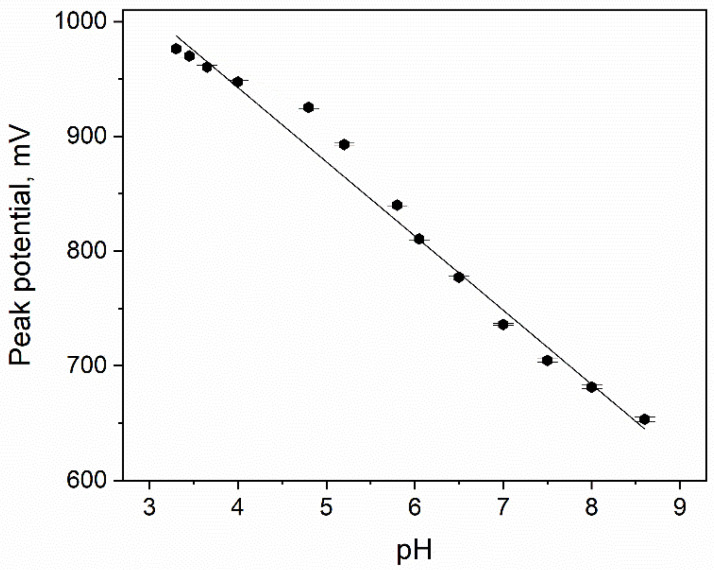
Dependence of pH of phosphate buffer (0.1 M) on CTZ peak potential.

To gather more information concerning the electrode process, the influence of pH on the CTZ peak potential was verified (Figure 5). The experiment was carried out in the 0.1 M phosphate buffer in the pH range of 3.3–8.6. The linear dependence was obtained and may be described by the following Equation (1):

(1) $$E_{p}=-64.7\;\mathrm{pH}+1201.3\;\mathrm{mV}\;r=0.991\;$$

A slope of the obtained dependence is close to the theoretical value (59 mV pH^−1^), which suggests that one proton per one electron is exchanged during the electrode reaction.

**Figure 6:**
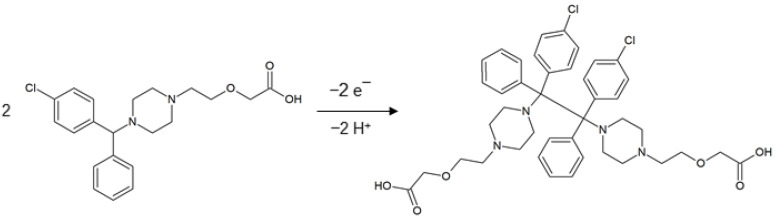
Proposed mechanism of CTZ electrode reaction [18].

After putting all the information together, it may be concluded that the CTZ electrode reaction involves two protons and two electrons. The possible mechanism of the electrode reaction is presented in the Figure 6. The proposed mechanism is consistent with the one described by P.R. Vernekar [18].

### 3.4. Supporting-Electrolyte Optimization

#### 3.4.1. Type of the Supporting Electrolyte

The choice of supporting electrolyte is an important part of developing a new voltammetric method. Selecting an appropriate environment ensures a stable signal and helps to increase the sensitivity of the method; therefore, an appropriate experiment was conducted. During all measurements, the CTZ concentration was equal to 10 µM, and the accumulation potential and time were equal to 300 mV and 10 s, respectively. The following potential electrolytes were tested: 0.1 M acetate buffer pH 4.5, 0.1 M phosphate buffer pH 7.0, 0.1 M hydrochloric acid, 0.1 M ammonia buffer pH 8.2, 0.1 M monopotassium phosphate, 0.1 M dipotassium phosphate. The highest peak current of CTZ (8.1 ± 0.2 µA) was obtained in 0.1 M phosphate buffer. Additionally, the peak was well-shaped and the peak to background ratio was favorable; therefore, further measurements were conducted in the electrolyte mentioned.

#### 3.4.2. Concentration of Phosphate Buffer

Another important aspect is optimization of the concentration of the supporting electrolyte. In this experiment, the CTZ signal (10 µM added) was measured in phosphate buffer (pH 7.0) with different concentrations. During all measurements, the accumulation potential and time were equal to 300 mV and 10 s, respectively. We have observed that for lower concentrations of phosphate buffer (0.025 M and 0.05 M), the background current is relatively high. This means that insufficient conductivity of the electrolyte was ensured, and a higher concentration must be applied. The electrolyte consisting of 0.1 M phosphate buffer gave good results, a level of background current was acceptable, and the CTZ signal was stable and high (8.2 ± 0.2 µA, RSD = 2.4%, *n* = 3); therefore, further measurements were conducted under such conditions.

### 3.5. DPV Parameters Optimization

Optimization of the DPV technique parameters allows us to improve sensitivity of the method. Therefore, in the experiment, each parameter was optimized. During the experiment, the CTZ concentration was equal to 10 µM, and the supporting electrolyte consisted of 0.1 M phosphate buffer (pH 7.0). Accumulation potential and time were equal to 300 mV and 10 s, respectively. The waiting and sampling times (t_w_ and t_s_) were tested in the range of 5 to 50 ms. Results show that in for both parameters, the lower the value, the higher the register CTZ signal. Nevertheless, for the lowest value from the tested range (5 ms), the signal was unstable; therefore, 10 ms was chosen as optimal for t_w_ and t_s_. The step potential E_s_ was optimized in the range of 1–6 mV. The experiment showed that the higher was the value of E_s_, and the higher was the CTZ signal; therefore, 6 mV was chosen as optimal. The last optimized parameter was the pulse amplitude, ΔE. Its value was tested in the range of 5 to 100 mV (positive and negative mode). The best results were obtained for 75 mV, and therefore, this value was used in further measurements.

### 3.6. Accumulation Potential and Time Optimization

The accumulation potential and time are characteristic parameters for the stripping voltammetry technique. The influence of both parameters on the CTZ signal was investigated. The experiment was carried out in 0.1 M phosphate buffer (pH 7.0) for two different CTZ concentrations—10 µM and 1 µM. In the case of the accumulation potential, no influence on the CTZ peak current or position was observed. An experiment with accumulation time has shown that there is also no influence of parameter on peak current and its position. However, we observed that when the accumulation step is not introduced, the CTZ signal is unstable—RSD = 12.1% (*n* = 3). When the short-accumulation time was applied (10 s), no improvement in peak current was observed, but the signal was stable (RSD = 3.2%, *n* = 3). It may be caused by adsorption of CTZ on the surface of the working electrode. Ensuring an appropriate time allows the electrode’s surface to saturate with analyte; therefore, a fluctuation in signal was not observed. Considering the results obtained, we decided to introduce a short accumulation step: 300 mV and 10 s to provide stability of the measurement.

### 3.7. Interferences Study

The developed voltammetric method for CTZ determination was designed for the measurement of different types of samples (pharmaceutical products, body fluids); thus, it is particularly important to study the influence of potential interferents on the analyte signal. During the experiment, the supporting electrolyte consisted of 0.1 M phosphate buffer (pH 7.0), accumulation potential and time were equal to 300 mV and 10 s, respectively, and CTZ concentration was equal to 10 µM. In the next step, the influence of different interferents on the CTZ signal was investigated. The following substances were tested: Fe(III), Pb(II), Zn(II), Cu(II), Al(III) (20 µM added), Mg(II), Ca(II) (150 µM added), SO_4_^2−^, NO_3_^−^, Cl^−^, CO_3_^2−^ (1000 µM added), citric acid, ascorbic acid, glucose (500 µM added), lactose monohydrate, aspartame, caffeine, uric acid (100 µM added), microcrystalline cellulose, magnesium stearate (5 mg added to 10 mL of electrolyte), and Triton X-100 (15 ppm added). Among the substances that had influence on CTZ were Fe(III) and Zn(II). The presence of 20 µM of Fe(III) caused a 20% drop in the CTZ peak current, and 20 µM of Zn(II) caused a 19% drop in the CTZ peak current. Another substance that caused deterioration of the measurement conditions was aspartame. A concentration equal to 100 µM caused a 24% decrease in the peak current. Triton added in a concentration of 15 ppm caused a 63% decrease in the CTZ peak current value. The remaining interferents had no or a negligibly small influence on the CTZ signal.

### 3.8. Analytical Performance

**Figure 7:**
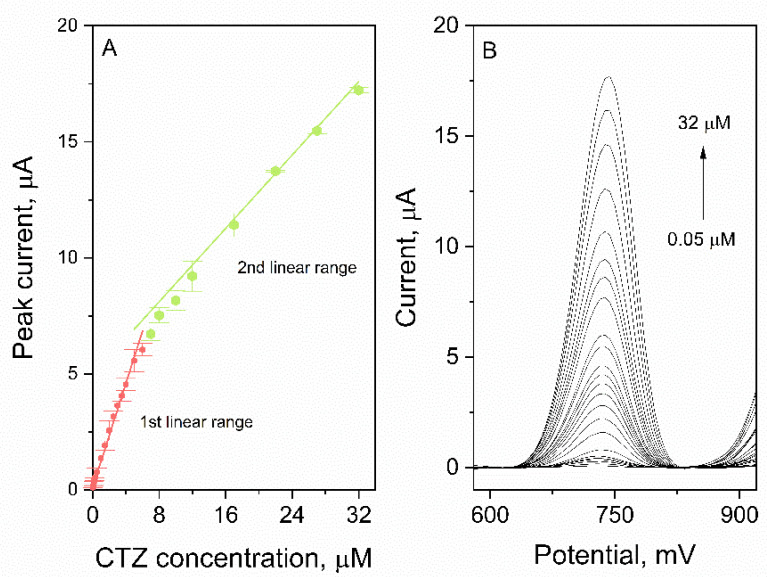
(**A**) CTZ calibrations with 2 linear ranges: 0.05–6 µM; 7–32 µM. (**B**) Voltammograms registered during calibration.

To verify the analytical performance of the developed method for CTZ determination, calibration was conducted. The experiment was carried out in optimized measuring conditions. The dependence of the CTZ peak current on its concentration in the electrochemical cell as well as in the corresponding voltammograms are presented in the Figure 7. As it may be observed, two linear ranges were identified: 0.05–6 µM (slope 1.12 ± 0.05 µA µM^−1^, intercept 0.09 ± 0.02 µA, *r* = 0.988); 7–32 µM (slope 0.40 ± 0.02 µA µM^−1^, intercept 4.9 ± 0.5 µA, *r* = 0.992). Based on the obtained data, the LOD and LOQ values were calculated (LOQ = 3 LOD; LOD = 3.3 s/b, where s—signal noise, b—slope of the calibration) and were equal to 14 nM and 42 nM, respectively. The comparison of the LOD value of our method with other voltammetric methods described in the literature is presented in the Table 1. To verify the usefulness of the developed method, the CTZ concentration in the real samples was determined. Pharmaceutical products in the form of tablets and body fluids (plasma, urine) were prepared according to the procedure described in Section 2.3 and measured using the standard addition method and the procedure is presented in Section 2.6. Before measurement, lyophilizate urine and plasma were diluted: 10×, 40× and 35×, 125×, respectively. The obtained results are presented in Table 2. The recoveries calculated based on the sample measurements were in the range of 97–115%, which suggests that the developed method for CTZ determination may be considered accurate.

## 4. Conclusions

A new electrochemical sensor for highly sensitive CTZ determination was developed. The proposed sensor exhibits good electrochemical properties and allowed us to improve the CTZ signal eight times in comparison to the bare GCE. The instrumental parameters of the voltammetric method and the measurement conditions were optimized for CTZ determination. Value of the LOD was calculated based on performed calibration and was equal to 14 nM. This is a good result in comparison with others presented in the literature, especially as our sensor is simple and fast in preparation and may be characterized by good repeatability (RSD = 3.4, *n* = 9) and reproducibility (RSD = 9.7%, *n* = 5). To verify the usefulness of the developed sensor and optimized method in real sample analysis, CTZ was determined in samples characterized by simple and complex matrices—pharmaceutical products and body fluids, such as urine and plasma. Recoveries calculated based on performed sample measurements were in the range of 97–115%, which is an acceptable result considering the complexity of the sample matrix; therefore, the developed method may be considered as accurate. The results obtained from the samples were also satisfactory and led us to the conclusion that our method could be used in routine laboratory practice.

## Figures and Tables

**Table 1:** Comparison of LOD values for CTZ obtained using voltammetric methods.

Type of WE	LOD, nM	Reference
CPE-MWCNTs-Pt	58.6	[14]
PLMCNTPE	170	[16]
Bent/CPE	59.1	[18]
CTABMCNTPE	270	[22]
RuTiO_2_-MWCNTs	3.1	[24]
MWCNT-GCE	70.7	[15]
chitosan/IL/MWCNTs/GCE	8	[17]
BiF-GCE	1.5	[23]
GCE	4500	[21]
GPE	160	[20]
BDDE	16	[19]
eCNF/CNT/NiCo-GCE	14	This work

CPE-MWCNTs-Pt—carbon paste electrode modified with multi-walled carbon nanotubes and Pt nanoparticles; PL CNTPE—poly(L-Leu) modified carbon nanotube paste electrode; Bent/CPE—carbon paste electrode with layered structured bentonite clay; CTABMCNTPE—cetyl trimethylammonium bromide modified carbon nanotube paste electrode; RuTiO_2_-MWCNTs-CPE—ruthenium-doped titanium dioxide nanoparticles and multi-walled carbon nanotubes modified carbon paste electrode; MWCNT-GCE—multi-walled carbon nanotubes modified glassy carbon electrode; chitosan/IL/MWCNTs/GCE—chitosan, ionic liquid and multi-walled carbon nanotubes modified glassy carbon electrode; BiF-GCE—bismuth film glassy carbon electrode; GPE—graphite pencil electrode; BDDE—boron doped diamond electrode.

**Table 2:** Results of sample measurements: tablets and urine.

Sample	CTZ Added, mg	CTZ Found ± s, mg (Recovery, %)
Allertec WZF ^a^	0	10.9 ± 0.7
	10	25.0 ± 0.9 (108)
	20	34 ± 3 (100)
Cirrus Duo ^b^	0	5.5 ± 0.2
	5	12.9 ± 0.1 (109)
	10	17.3 ± 0.6 (110)
Sample	CTZ added ± s, µM	CTZ found ± s, µM (recovery, %)
Urine diluted 40×	0	ND
	1	1.1 ± 0.1 (108)
	2	2.3 ± 0.3 (110)
Urine diluted 10×	0	ND
	3	3.1 ± 0.2 (103)
	6	6.9 ± 0.5 (110)
Plasma diluted 125×	0	ND
	1	1.2 ± 0.1 (115)
	2	2.3 ± 0.3 (113)
Plasma diluted 35×	0	ND
	3	3.3 ± 0.2 (109)
	6	5.8 ± 0.5 (97)

ND—not detected; ^a^ producer declares 10 mg of CTZ in tablet; ^b^ producer declares 5 mg of CTZ in tablet.

## Data Availability

The data presented in this study are available on request from the corresponding author.

## Supplementary Materials

The following supporting information can be downloaded at: https://www.mdpi.com/article/10.3390/ma15103648/s1, Figure S1: EDS spectra of eCNF/CNT/NiCoNP nanocomposite (the lines from Cu originate from used copper TEM grid, while the oxygen is coming from the carbon-oxygen functional groups and absorbed oxygen and water molecules), Figure S2: HRTEM image showing the NiCo particles.
