# Supplementary material for: New Electrochemical Sensor Based on Hierarchical Carbon Nanofibers with NiCo Nanoparticles and Its Application for Cetirizine Hydrochloride Determination
Source: Materials (Basel). 2022 May 20;15(10):3648. doi: 10.3390/ma15103648 (PMC9147852; doi:10.3390/ma15103648)
Supplement: Supplementary file 1 [file materials-15-03648-s001.zip › materials-1661835-supplementary.pdf]

Supporting Information:

New electrochemical sensor based on hierarchical carbon nanofibers with NiCo nanoparticles and its application for cetirizine hydrochloride determination

Anna Górska, Marcel Zambrzycki, Beata Paczosa-Bator, Robert Piech*

Faculty of Materials Science and Ceramics, AGH University of Science and Technology, Al. Mickiewicza 30, 30-059, Krakow, Poland

E-mail: rpiech@agh.edu.pl

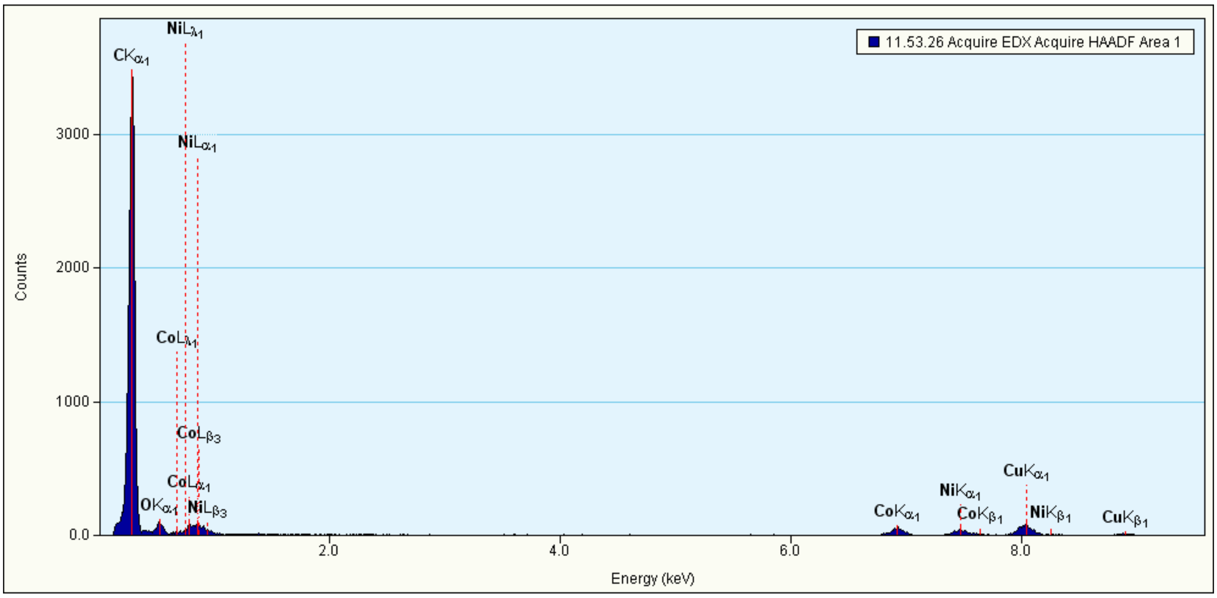

Figure S1. EDS spectra of eCNF/CNT/NiCoNP nanocomposite (the lines from Cu originate from used copper TEM grid).

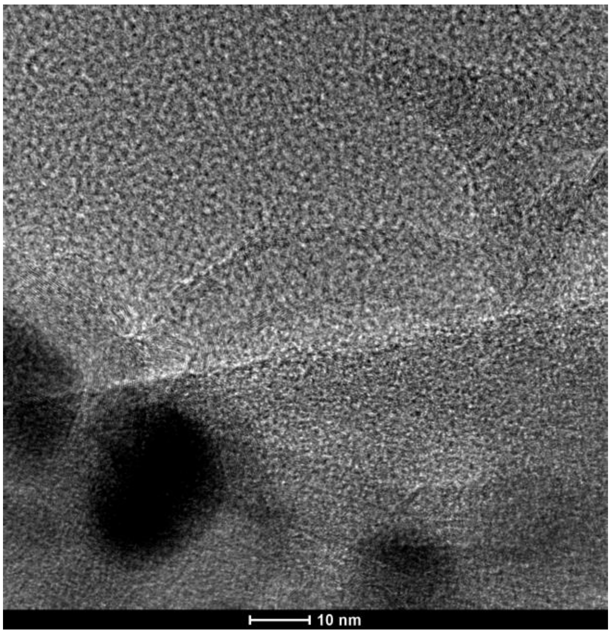

Figure S2. HRTEM image showing the NiCo nanoparticles embedded on the surface of eCNF.
